# Endopolyploidy as a potential alternative adaptive strategy for *Arabidopsis* leaf size variation in response to UV-B

**DOI:** 10.1093/jxb/ert473

**Published:** 2014-01-27

**Authors:** Vasilis C. Gegas, Jason J. Wargent, Edouard Pesquet, Emma Granqvist, Nigel D. Paul, John H. Doonan

**Affiliations:** ^1^John Innes Centre, Norwich Research Park, Norwich NR4 7UH, UK; ^2^Lancaster Environment Centre, Lancaster University, Lancaster LA1 4YQ, UK; ^3^IBERS, Aberystwyth University, Aberystwyth SY23 2AX, UK

**Keywords:** Abiotic stress, *Arabidopsis*, endopolyploidy, natural variation, organ development, UV-B.

## Abstract

The extent of endoreduplication in leaf growth is group- or even species-specific, and its adaptive role is still unclear. A survey of *Arabidopsis* accessions for variation at the level of endopolyploidy, cell number, and cell size in leaves revealed extensive genetic variation in endopolyploidy level. High endopolyploidy is associated with increased leaf size, both in natural and in genetically unstructured (mapping) populations. The underlying genes were identified as quantitative trait loci that control endopolyploidy in nature by modulating the progression of successive endocycles during organ development. This complex genetic architecture indicates an adaptive mechanism that allows differential organ growth over a broad geographic range and under stressful environmental conditions. UV-B radiation was identified as a significant positive climatic predictor for high endopolyploidy. *Arabidopsis* accessions carrying the increasing alleles for endopolyploidy also have enhanced tolerance to UV-B radiation. UV-absorbing secondary metabolites provide an additional protective strategy in accessions that display low endopolyploidy. Taken together, these results demonstrate that high constitutive endopolyploidy is a significant predictor for organ size in natural populations and is likely to contribute to sustaining plant growth under high incident UV radiation. Endopolyploidy may therefore form part of the range of UV-B tolerance mechanisms that exist in natural populations.

## Introduction

In plants, the dramatic increase in cell size that occurs during the post-proliferative phase is often coupled with an increase in nuclear DNA content through the process of endoreduplication (Gutierrez, 2009). Endoreduplication is a specialized mode of cell cycle that allows extra rounds of DNA replication to occur without intervening cell divisions and it is often closely associated with specific cell types, organs, and developmental stages (Galbraith *et al.*, 1991; Sugimoto-Shirasu and Roberts, 2003). In animals, endoreduplication has a recognized role in driving body size (Flemming *et al.*, 2000) or in maintaining tissue and organ growth in response to exogenous stresses, such as regeneration of damaged liver and cardiomyocytes (Lee *et al.*, 2009).

Although, endopolyploidy is widespread among plant taxa (Nagl, 1976; Galbraith *et al.*, 1991; Barow, 2006), its role in development and adaptive significance are still hotly debated (Gutierrez, 2009). Endosperm, formed as a result of double fertilization and effectively a genetic cul-de-sac, tends to display high levels of endoreduplication. Other large terminally differentiated cells, for example, xylem precursors also endoreduplicate in many species but not in others. In developing leaves of *Arabidopsis thaliana*, endoreduplication is also associated with the onset of cell differentiation (Dewitte *et al.*, 2003) and it is positively correlated with an increase in cell size (Melaragno *et al.*, 1993) and rapid leaf growth (Donnelly *et al.*, 1999). Natural variants with increased endopolyploidy have been associated an 8-bp insertion in the 3′-UTR of the cyclin D5 gene (Sterken *et al.*, 2012) and manipulation of a number of related cyclin genes can be used to alter the progression of endoreduplication in various tissues (Dewitte *et al.*, 2007).

Stress tolerance has been suggested as an important functional role for endoreduplication within plant development (Barow and Meister, 2003; Adachi *et al.*, 2011). Moreover, endoreduplication may form an important component of plant response to ultraviolet radiation, particularly UV-B radiation (290–320nm). Hase *et al.* (2006) showed that the *UV-B-insensitive 4* (uvi4) mutant underwent an additional round of endoreduplication in hypocotyl cells and that both *uvi4* plants and tetraploid *Arabidopsis* were relatively insensitive to UV-B treatment. Endocycle responses to UV-B radiation are regulated by the UV-B photoreceptor UV RESISTANCE LOCUS 8 (UVR8) (Wargent *et al.*, 2009) and an endocycle regulator, atypical E2F transcription factor DEL1, has been linked to establishment of UV-B tolerance via control of the type-II cyclobutane pyrimidine dimer-photolyase DNA repair gene PHR1 (Radziejwoski *et al.*, 2011). However, the possible role of the endocycle in sustaining plant growth in response to UV-B radiation in natural populations has been poorly characterized.

Using both natural variants and D-cyclin T-DNA mutants, this study demonstrates that endopolyploidy is a highly significant explanatory cellular factor that correlates with the variation of organ size in natural populations, particularly in response to UV-B radiation, and may, therefore, be of adaptive significance in climates with high solar irradiation.

## Materials and methods

### Plant material and growth conditions

*Arabidopsis* accessions and mutants were obtained from the Nottingham *Arabidopsis* Stock Centre. The Kondara-Br0 and Ler-Kondara recombinant inbred lines were as previously described (el-Lithy *et al.*, 2006; O’Neill *et al.*, 2008). Unless otherwise stated, plants were grown under long days (16/8 light/dark cycle) on soil. All the analyses were performed on the fifth rosette leaf at day 15 post initiation. Under these conditions, leaves had reached maturity by that stage. Day of leaf initiation (day 0) was defined as when the leaf was visible under ×10 magnification.

### Flow cytometry of *Arabidopsis* leaves

The tissue chopped finely with a razor blade in 500 μl extraction buffer (Partec, Germany), filtered through a 30-μm mesh (Partec), and 1ml of Cystain UV staining solution was added. Endopolyploidy analysis was performed with a PAS II Ploidy analyser (Partec) using an arc-lamp. In each run, 20 000 events were counted at an average speed of 50 events s^–1^. All the data was acquired on a logarithmic amplification (log3) scale unless otherwise stated. Endoreduplication index (EI) was calculated as described before (Barow and Meister, 2003).

### Cytology

Leaves were harvested and fixed immediately in ethanol/glacial acetic acid (1:1) for 12h at 4 °C. After fixation, leaves were dehydrated in an ethanol series (50, 70, 80, 100% for 20min each). Subsequently, the leaves were immersed in a clearing solution (chloral hydrate/glycerol/H_2_O (8:2:1). Samples were observed with a Nikon MicroPhot-SA microscope using DIC optics and images were captured with a Nikon CoolPix 990 digital camera. Six images per leaf were taken (i.e. three consecutive images per lamina side). Cell density was determined by counting all the cells included in a fixed image area (six images per leaf; five leaves per genotype). The total number of cells per leaf (referred to as cell number) was then calculated from the leaf area measurements. Statistical analysis of the results was performed using SPSS version 12.0.1 (SPSS, Chicago, Illinois, USA).

### Hierarchical clustering and principal component analysis

Raw data processed using hierarchical clusterization explorer (HCE) version 3.5 (Seo and Shneiderman, 2002) and SIMCA-P+ version 10.0 (Umetrics, Sweden) for hierarchical clustering and principal component analysis (PCA), respectively. For the extraction of principal component (PCs), the correlation matrix extraction method was used. Only the factors with an eigenvalue ≥1 according to Kaiser’s criterion were retained (Jolliffe, 2002). Each principal component (PC) was defined by an *R* ^2^ explanation value and a specific loading arrangement defining the relationship between each category subset of the analysed data. Closest PCs resulting from different PCA were defined using linear correlation.

### Quantitative analyses

Prior to any quantitative analysis, the symmetry of the distribution and the normality of the observed data were tested. QTL mapping on both transformed and untransformed data gave similar results (data not shown). Pierson and Spearman rho correlations between traits were similar. The MapQTL version 5.0 (Van Ooijen, 2004) was used for the analysis of the quantitative data. A genome-wide threshold LOD value for significant QTL was set at 2.4 and 2.5 (*P* < 0.05) for the Kondara-Br0 and Ler-Kondara RIL populations, respectively, by performing 10 000 permutations of the original data (Churchill and Doerge, 1994; Doerge and Churchill, 1996). The software Epistat (Chase, 1997; http://527270.sites.myregisteredsite.com/epistat.htm) was used to identify and test interactions between pairs of QTL. The automated search routine was performed to search for all pairwise interactions, having the stringent cut-off value of 6 as an initial likelihood ratio threshold for significant interactions (~*P* = 0.0005 according to Chase, 1997). All interactions where the markers were separated by <50 cM were removed to control for linkage effects (Malmberg and Mauricio, 2005). Statistical significance for the detected interactions was established by Monte Carlo simulations (1 000 000 trials). The threshold *P*-value for significant interactions was derived by dividing the required *P*-value (0.01) by *n*(*n* – 1)/2, where *n* = number of chromosomes (Malmberg and Mauricio, 2005). Therefore, *P*-value was set at the conservative level of 0.001.

### Environmental data and UV irradiation

The relationships between plant traits and environmental variables were determined using mean temperature data from the VNAT database (http://publiclines.versailles.inra.fr) and UV-B data for the appropriate 0.5° grid square from the UV climatology based on ozone measurements made by the GOME instrument carried by the ERS-2 satellite (http://www.temis.nl/uvradiation/GOME). The relationships presented are for mean annual erythemally weighted UV-B radiation (McKinlay and Diffey, 1987), but relationships were broadly similar using maximum UV or the alternative DNA-weighting function (Setlow *et al.*, 1993).

All analyses were conducted using linear multiple regressions in PASW statistics version 17.0 (SPSS).

UV radiation treatments were applied in a similar method to that used previously by this study group (Wargent *et al.*, 2009). Selected lines were stratified as described earlier, but were then transferred into a group of three controlled environment growth cabinets (Microclima 1750, Snijders Scientific, Tilburg, Netherlands), which contained a series of PAR sources: (20× Sylvania Luxline Plus, FH024W/T5 840, 550mm; 10× Sylvania Luxline Plus, FH054/T5 840, 1150mm; 6× Sylvania BriteGro, F58W/T8 2023, 1514mm; all CEC Technology, Glasgow, UK), delivering a PAR flux of 300±20 μmol m^–2^ s^–1^. The conditions for growth were 10/14 light/dark cycle (both PAR and UV), 21/18±2 °C, and 60% relative humidity. Supplementary UV-B exposure commenced prior to fifth rosette leaf initiation and was provided by three UV-B tubes (Q-Panel 313, Q-Panel Laboratory Products, Bolton, UK) wrapped in 0.13mm cellulose diacetate film (Clarifoil, Courtaulds, Derby, UK) in order to exclude all wavelengths below 290nm. Zero UV-B control conditions were provided by wrapping one end of each supplementary UV-B tube with a commercial-grade UV-opaque polyethylene film (Lumivar, BPI Visqueen, Ardeer, Scotland) and in addition, a sheet of clear polyester (Lee Filters, Andover, UK) was fitted between chamber treatment regions in order to prevent wavelengths below 320 nm reaching the control area of the chamber bench. Plants were routinely moved between cabinets to avoid any positional/microclimatic bias. All UV treatments were quantified using a double monochromator scanning spectroradiometer (model SR991-v7, Macam Photometrics, Livingston, UK). UV treatments were determined using the generalized plant action spectrum (Caldwell, 1971), providing a UV-B dose of 10 kJ m^–2^ day^–1^. For quantification of UV-B-absorbing compounds, the method followed that of Gonzalez *et al.* (1996).

## Results

### Natural variation in endopolyploidy

Size variation between different plant and animal taxa is generally attributed to cell number differences. However, other factors, such as cell size and endopolyploidy, can contribute to variation in size within taxa or even within species (Edgar and Orr-Weaver, 2001). Characteristically, cell size and endopolyploidy were shown to drive organ size in nematodes (Flemming *et al.*, 2000; Lozano *et al.*, 2006) and *Drosophila* (Edgar and Orr-Weaver, 2001), respectively. The present study hypothesized that a variety of cellular mechanisms might also account for the natural variation in leaf size apparent in *Arabidopsis* accessions and defined the natural variation for three cellular parameters related to organ size—cell number, cell size, and somatic endopolyploidy—in fully matured leaves (Supplementary Table S1, available at *JXB* online) from Col0, a widely used laboratory reference strain believed to originate ultimately from Germany, and from a collection of geographically diverse accessions that have been used to create genetically unstructured mapping populations (el-Lithy *et al.*, 2006; O’Neill *et al.*, 2008).

**Fig. 1.**
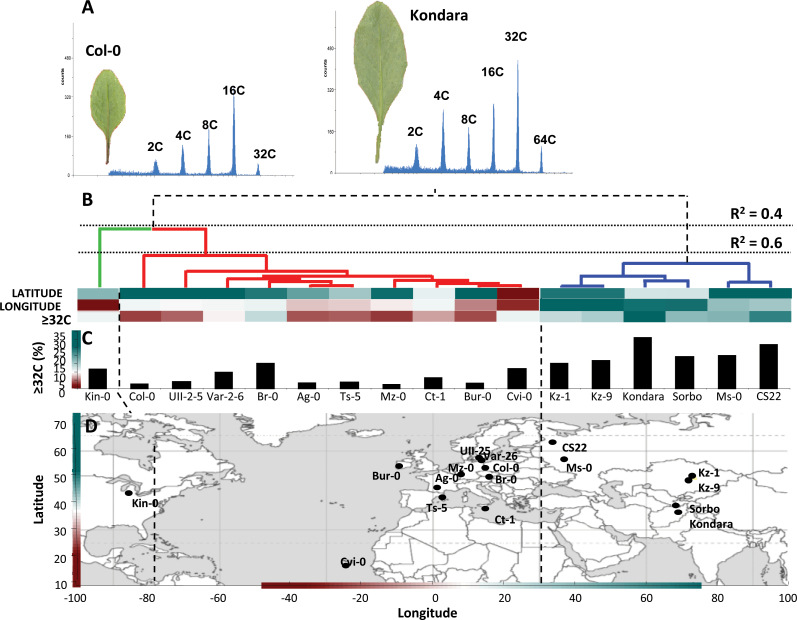
Somatic endopolyploidy varies in the leaves of *Arabidopsis* accessions. (A) Endoreduplication profile in the fifth leaf of Col-0 and Kondara. The fifth rosette leaf at maturity is featured (adaxial side) at the left of each graph (see also Supplementary Table S1 available at *JXB* online). (B) Hierarchical clustering for the ≥32C ploidy and corresponding geographical coordinates (longitude, latitude) at the original sites of collection. Minimum similarity for cluster partition is given as *R* ^2^ values. Different clusters within the cladogram are depicted in different colours: blue, Central Asia/Russia; red, Europe; green, America. Longitudinal/latitudinal positioning and the endopolyploidy values are depicted by colour-coded gradient scale (refer to Figs. C and D). (C) Distribution of the high endopolyploidy fragments (≥32C) of *Arabidopsis* accessions relative to their geographic origin. Values are mean of three biological replicates expressed as percentage of the total nuclei counted. (D) Geographic origins of the *Arabidopsis* accessions (see also Supplementary Table S1 available at *JXB* online).

The endopolyploidy profile was determined by flow cytometric analysis of nuclei isolated from the fifth rosette leaf at maturity (15 d post initiation; Fig. 1A) taken from plants grown on soil. The accessions vary considerably in the extent of endopolyploidy (Fig. 1A, C; see also Supplementary Table S1 available at *JXB* online), most notably in the higher ploidy fractions: i.e. 32C (range 2.15–25.4%) and 64C (0–5.6%). Natural variation was also evident for the other cellular parameters across the accessions studied (Supplementary Table S1 available at *JXB* online). with minimum cell density of 122.0 cells mm^–2^ in the Asian accession Kondara (Tajikistan) to a maximum 192.0 cells mm^–2^ in Mz-0 (Germany) (mean 159.0±18.4 cells mm^–2^). The mean cell size across the accessions (Supplementary Table S1 available at *JXB* online) was 6514±822 μm^2^ (min, 5282 μm^2^ Mz-0; max, 8395 μm^2^ Kondara). Hierarchical clustering (Fig. 1B) identified two main clusters of accessions (*R* ^2^ = 0.6, *P* < 0.05) that showed significant differences (*t* = –6.67, *P* < 0.01) in the level of ≥32C ploidy (cluster1 mean_≥32C_, 5.5%; cluster 2 mean_≥32C_, 22.1%) and broadly reflected the geographic origins of the accessions (Fig. 1D). The clusters also differed significantly for the related traits of cell density (*t* = 7.12, *P* < 0.01), cell size (*t* = –7.44, *P* < 0.01), and leaf size (*t* = 5.03, *P* < 0.01).

**Fig. 2.**
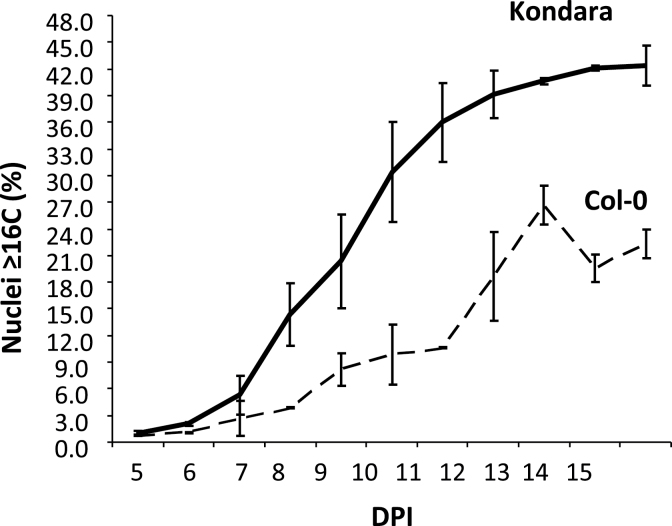
Advanced progression of successive endocycles in Kondara. The developmental series corresponds to days post initiation (DPI) of the fifth rosette leaf. Values are mean percentage ± standard deviation of three biological replicates of the endoreduplication fractions that correspond to 16C and above (see also Supplementary Fig. S1 available at *JXB* online). Time points between 8 and 15 DPI are significantly different in pairwise comparisons between Kondara and Col-0 (two-tailed *t*-test, *P* < 0.05).

To investigate the cellular mechanisms underlying the differences in the endopolyploidy profile, this work performed a time-course analysis of endoreduplication in two representative accessions (Fig. 2). Kondara, a high endopolyploidy accession, shows more advanced progression through consecutive rounds of endoreduplication compared with Col-0. As early as 8 d post initiation, Kondara had approximately 3-fold higher endopolyploidy (≥16C) compared with Col-0 (Fig. 2), which may be attributed to a faster succession of endocycles (i.e. 8C to 16C, 16C to 32C; Supplementary Fig. S1 available at *JXB* online). Kondara therefore sustains a much higher ploidy level throughout leaf development.

### Endopolyploidy variation correlates with leaf size variation

**Fig. 3.**
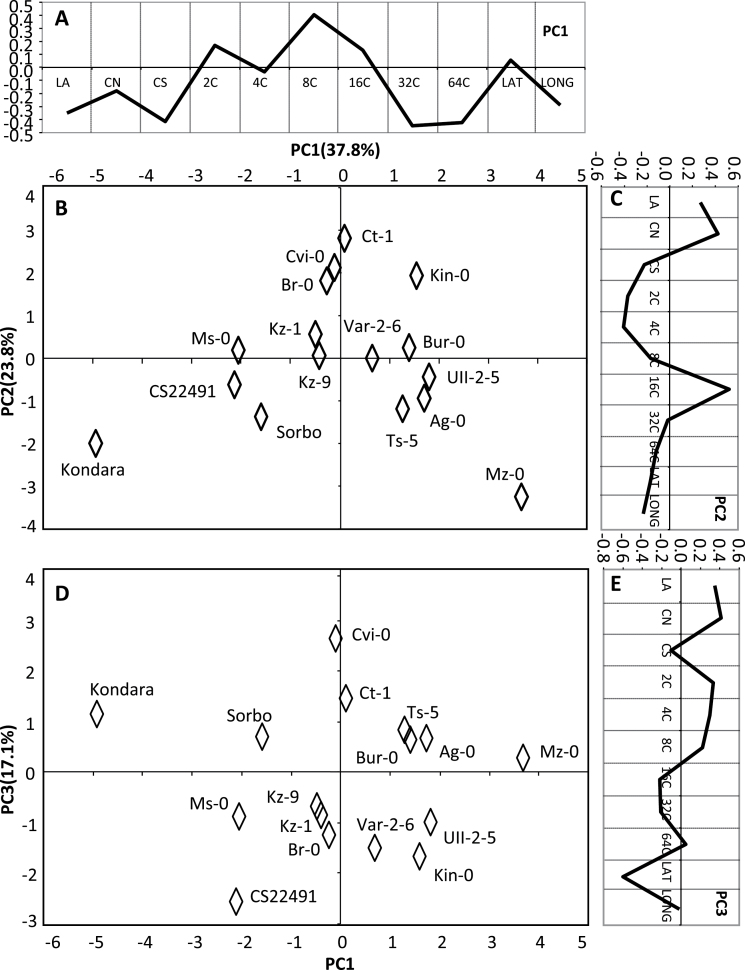
A phenotypic model for variation in leaf size and cellular parameters. The model defines cell number and high ploidy as significant factors for organ size variation. For example, Kondara and Ct-1 both reach similar leaf size but by increased high ploidy and cell number, respectively. In contrast, reduced ploidy acts as a limiting factor for leaf size in Mz-0. Three principal components (PC1–PC3) account for 78.7% of the variation in the *Arabidopsis* accessions. (A, C, E) Probability loadings of PC1, PC2, and PC3. (B) PCA sample distribution for PC1 versus PC2. (D) PCA sample distribution for PC1 versus PC3. Percentage of variation explained by each PC is given in parentheses. CN, cell number; CS, cell size; LA, leaf area; LAT, latitude; LONG, longitude (see also Supplementary Tables S1 and S2 available at *JXB* online).

Principal component analysis was performed to identify the pattern of association, and possible interdependence, between the different cellular and morphometric traits. PCA does that by identifying orthogonal directions, namely PCs, along which the trait variance is maximal (Jolliffe, 2002). The PCA model shows that 78.7% of the variation in the *Arabidopsis* accessions studied was captured by three principal components that factor both the geographical dispersion and differences at the cellular parameters (Fig. 3; see also Supplementary Table S2A available at *JXB* online). Most importantly, variation at the higher endopolyploidy levels was identified as a highly significant and hitherto unknown explanatory factor for differences between the accessions (Fig. 3A, B). In PC1 (37.8% variation explained), 32C and 64C are the major explanatory factors (*R* ^2^ = 0.801 and 0.822, respectively) and they are positively associated with cell size and leaf area (Fig. 3A, B). Cell number is also positively associated with leaf area in PC2 and PC3 (Fig. 3C–E), which is consistent with the recognized role of cell number in sustaining organ growth (Gonzalez *et al.*, 2010).

**Fig. 4.**
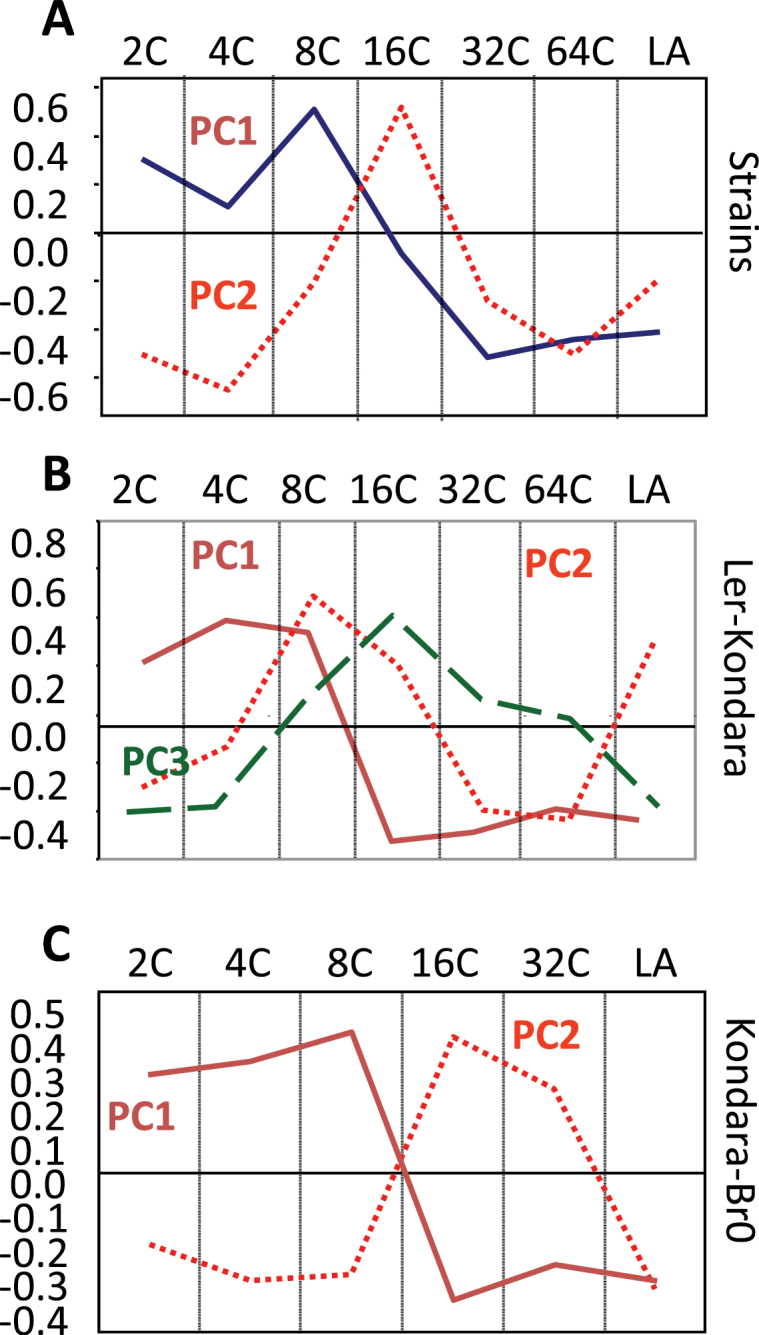
Phenotypic model for variation in endopolyploidy and leaf area in *Arabidopsis* accessions is analogous to that of the two mapping populations. Probability loadings for the principal components extracted for the *Arabidopsis* accessions (A) and the Ler-Kondara (B) and Kondara-Br-0 (C) RIL populations: blue solid line, PC1; red dotted line, PC2; green dashed line, PC3 (see also Supplementary Table S2 available at *JXB* online).

Confounding population structure is extensive in *Arabidopsis* natural accessions (Aranzana *et al.*, 2005) and this may cause spurious correlations between traits, especially if the traits show clinal variation, as is the case with variation in endopolyploidy. Therefore, this study examined two unstructured populations derived by experimental crosses between different *Arabidopsis* accessions. Two recombinant inbred line (RIL) populations, Kondara-Br-0 (O’Neill *et al.*, 2008; 94 RILs) and Ler-Kondara (el-Lithy *et al.*, 2006; 127 RILs) were analysed for the traits of endopolyploidy and leaf size. There were significant differences between the parental lines (i.e. Ler and Br-0 compared with Kondara) for the traits of leaf size, 32C, and 64C (two-tailed *t*-test, *P* < 0.001). In both RIL populations, significant positive correlations were observed between leaf size and the higher endopolyploidy fragments (i.e. 16C, 32C, 64C; Supplementary Table S3A, B available at *JXB* online), whereas the lower endopolyploidy fragments (i.e. 2C, 4C, 8C) were inversely correlated with leaf size (Supplementary Table S3A, B available at *JXB* online). This observation is in agreement with the PCA on the *Arabidopsis* accessions where leaf size is positively associated with higher endopolyploidy. PCs extracted from both RIL populations have analogous organization with the principal components of the accessions (Fig. 4A). Noticeably PC1 is common to both populations and shows a strong positive association between high endopolyploidy and leaf size, similarly to PC1 in the accessions (Fig. 4B, C).

### Genetic basis of variation in endopolyploidy

**Fig. 5.**
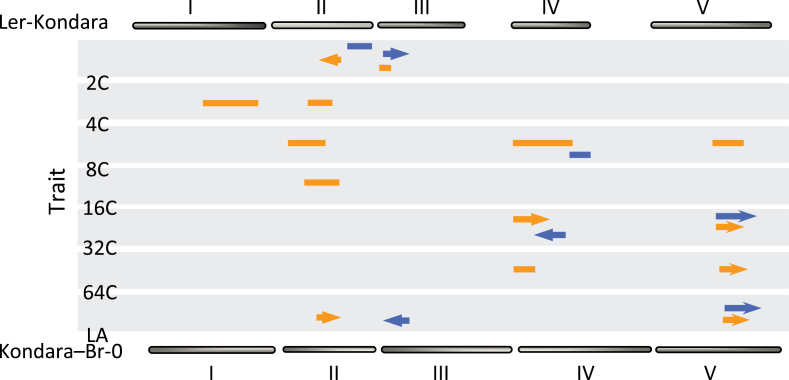
Genetic structure underlying the natural variation for endopolyploidy and leaf size in *Arabidopsis*. Schematic representation of co-segregated QTL for endopolyploidy and leaf area (LA) identified in the Ler-Kondara (orange bar) and Kondara-Br-0 (blue bar) populations. The length of each bar denotes 2-LOD confidence interval for the QTL on the corresponding linkage map of the RIL populations (see also Supplementary Table S5 available at *JXB* online). The direction of the allelic effects for the main co-segregating QTL is indicated by arrowheads (see also Supplementary Fig. S4 and Supplementary Tables S4 and S5 available at *JXB* online).

The phenotypic model linking variation in endopolyploidy with variation in leaf size described here suggests that these traits are under the control of common genetic components. To address this question, this study undertook quantitative approaches to identify the genetic architecture of natural variation in endopolyploidy level and leaf size. Broad-sense heritability (the proportion of variation attributed to genetic effects) was moderate to high for all traits, ranging in the Ler-Kondara population from 0.65 to 0.83 (Supplementary Fig. S2A-G available at *JXB* online) and in the Kondara-Br-0 from 0.58 to 0.86 (Supplementary Fig. S3A-F available at *JXB* online). In agreement with the extensive transgressive segregation (the emergence of extreme phenotypes in a segregating population, which was apparent for most of the traits studied; Supplementary Figs. S3 and S4 available at *JXB* online), several quantitative trait loci (QTL) with dispersed effects between the parents were identified (Fig. 5; see also Supplementary Table S4 available at *JXB* online). The significant associations identified between high endopolyploidy and leaf area are consistent with the presence of co-segregating QTL with the same or opposite allelic effect (Supplementary Table S4 available at *JXB* online). QTL for the 32C fraction co-segregate with the leaf area QTL (Fig. 5) and have the same allelic effect in both mapping populations (Supplementary Fig. S4E-H available at *JXB* online) with the Kondara allele increasing both the 32C fraction and the leaf area. In contrast, there is an opposing allelic effect between the overlapping QTL for the 2C fraction and leaf area (Fig. 5; see also Supplementary Fig. S4A-D available at *JXB* online), again consistent with the idea that high endopolyploidy is a driver of increased size.

Further pairwise marker analysis in both populations identified several epistatic interactions (Chase, 1997) for the control of endopolyploidy and leaf size (Supplementary Table S5 available at *JXB* online), indicating that the genetic architecture underlying these quantitative traits represents a network of additive QTL (that are common between the different mapping populations) and interacting QTL, with some of them involved in both additive and epistatic interactions. Epistatic interactions are often considered important components of natural variation both in plant (Malmberg *et al.*, 2005; Malmberg and Mauricio, 2005) and animal species (Shook and Johnson, 1999), especially for fitness or fitness-related traits. The results presented here demonstrate that epistatic effects have a significant role in the variation for endopolyploidy differences between the parental accessions.

### Endopolyploidy sustains growth under high UV radiation

Given the recognized role of endoreduplication in maintaining organ growth in animals, when exogenous stresses preclude or restrict cell proliferation (Lee *et al.*, 2009), this work hypothesized that an analogous model might also exist in plants and thus investigated whether the variation in endoreduplication found in *Arabidopsis* is associated with any particular climatic factors. Using climate data associated with the original collection sites of *Arabidopsis* accessions, temperature (obtained from the VNAT database) and solar UV-B radiation (derived from the GOME instrument carried by the ERS-2 satellite) were identified as the main predictors for variation in endopolyploidy (regression model; F = 8.704, *P* = 0.003) across the *Arabidopsis* accessions. These two factors together explained 55% (*P* = 0.003) of the variation in high endopolyploidy (≥32C). High endopolyploidy increased significantly with increasing UV radiation (*P* = 0.002) and with decreasing mean temperature (*P* = 0.001). Other climatic variable tested (including monthly precipitation, cloud cover, and solar radiation) did not provide significant explanation.

**Fig. 6.**
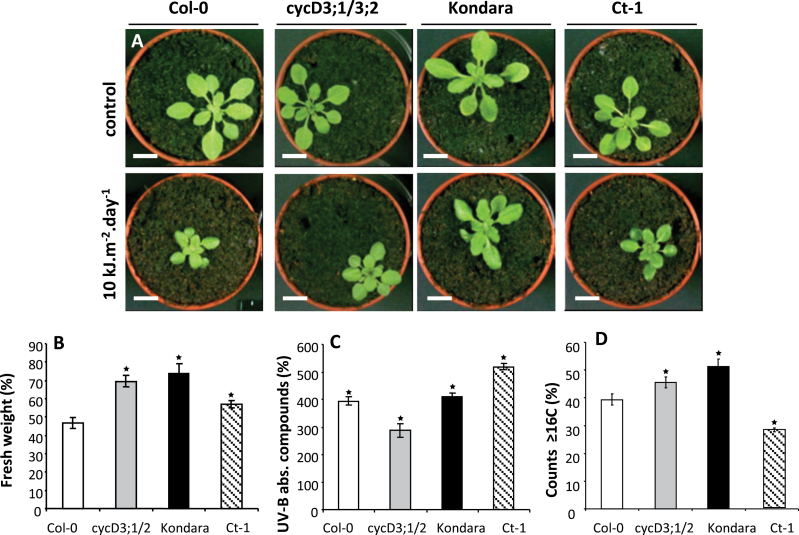
High endopolyploidy levels confer differential response to solar UV-B radiation in natural *Arabidopsis* accessions. (A) Rosettes of 30-d-old plants grown with (top) or without (bottom) supplementary UV-B radiation (10 kJ m^–2^ d^–1^). Bars, 1cm. (B) Fresh weight of whole rosettes after 16 d of exposure to UV-B (10 kJ m^–2^ d^–1^). (C) UV-B absorbing compounds induced after 16 d of exposure to UV-B. Values in B and C are mean percentages ± standard deviation of the mock plants. (D) Mean percentages ± standard deviation of the endopolyploidy fractions that corresponded to 16C and above (≥16C). Asterisks indicate significant differences with Col-0 (*P* < 0.01 after pairwise comparison (2-tailed *t*-test); for two-way ANOVA comparisons see Supplementary Table S6 available at *JXB* online).

UV-absorbing secondary metabolites (referred to hereafter as pigments) are generally considered to act as a ‘sunscreen’ (Jenkins, 2009), but it has recently been suggested that endopolyploidy also contributes to UV protection (Wargent *et al.*, 2009). To experimentally test this prediction, three accessions with contrasting levels of endopolyploidy and pigment induction were exposed (Fig 6C, D) to high but environmentally relevant (10 kJ m^–2^ d^–1^) UV-B radiation from before initiation of the fifth leaf until maturity. Col-0 was used as the baseline (‘normal’ for both endopolyploidy and pigment induction) and compared responses with Ct-1 (low endopolyploidy, high pigment induction) and Kondara (high endopolyploidy but normal pigment induction). As expected (Jansen *et al.*, 2010), UV-B reduced plant growth in all three accessions (Fig. 6A, B; see also Supplementary Table S6 available at *JXB* online), with Col-0 being the most sensitive (Fig. 6B). The relative UV tolerance of Ct-1 can be attributed to the high induction of pigments (Fig. 6C), which typically acts as a key response to UV radiation in many plant species. On the other hand, the enhanced tolerance exhibited by Kondara cannot be explained by upregulation of pigments, since pigment levels are induced to a similar degree both in Kondara and Col-0 by UV-B (Fig. 6C), but may be due instead to the high endopolyploidy.

To test if increased endopolyploidy could provide UV tolerance, this work examined the response of mutants with altered endopolyploidy. Loss of cyclin D3 genes, which control the switch between mitosis and endocycle during leaf growth (Gutierrez, 2009), results in elevated endopolyploidy (Dewitte *et al.*, 2007). The double loss-of-function mutant, *cycd3;1/3;2*, despite the low levels of induced pigments (Fig. 6C), is as tolerant to UV as Kondara (Fig. 6B,) indicating that artificially induced endopolyploidy (Fig. 6D) in an otherwise Col-0 background is sufficient to sustain growth under high UV-B radiation. Interestingly, constitutive pigmentation (i.e. in the absence of UV treatment) was lower in Kondara and Ct-1 compared with both Col-0 and *cycd3;1/3;2* (UV absorbing compounds g^–1^ FW^–1^: *cycd3;1/3;2* 1.28 > Col-0 1.04 > Ct-1 0.80 > Kondara 0.64).

## Discussion

Size control in the multicellular organs of both animals and plants poses a longstanding biological question that remains unsolved, mainly due to the complex regulation at cellular, organ, and whole-organism level (Cook and Tyers, 2007). Cell number has traditionally been seen as the main determinant for organ size and these two traits are highly associated in many plant and animal species (Conlon and Raff, 1999). Indeed, in plants, variation in the size of organs, such as tomato fruit (Frary *et al.*, 2000) and rice grains (Song *et al.*, 2007; Shomura *et al.*, 2008), has previously been attributed to differences in cell number. As in plants, endopolyploidy can also be a driver of organ growth in animals (Flemming *et al.*, 2000; Lozano *et al.*, 2006) and it can sustain organ size homeostasis in response to external stress (Lee *et al.*, 2009).

Endoreduplication plays a prominent and general role in the development of many organs in *Arabidopsis* and, in leaves, it is more or less tightly coupled to cell expansion depending on cell type (Cookson *et al.*, 2006). However, there are few reports relating endoreduplication and cell expansion in the leaves of many other species, including most grasses and major cereals. To dissect the genetic basis of this relationship, this work treated each level of endopolyploidy as a separate trait and asked which, if any, regions of the genome contributed to the observed variation. This analysis suggests that there are there at least three distinct genetic control mechanisms, at least two of which (2C and 32C/64C) colocate with loci that regulate leaf area. Leaf area and the proportion of nuclei with a 2*n*/4*n* ploidy level are antagonistic traits in both populations, although the position of the QTL pairs differ. In Ler, the QTL pair lies on chromosome II, overlapping the *Erecta* locus. This interpretation agrees with previous studies that have shown reduction in ERECTA function leads to prolonged cell proliferation, reduced cell expansion, and consequential reduction in leaf expansion (Tisne *et al.*, 2011). In the Br0 × Kondara population, a significant pair of antagonistic QTL colocate on chromosome III, suggesting different mechanisms in different accessions. In both cases, an increased portion of 2C nuclei is associated with decreased leaf area. Conversely, both populations reveal strong colocating QTL with similar effect on leaf area and high ploidy (32C in Br0 × Kon and both 32C and 64C in the other population), strongly supporting the notion that increased ploidy is very closely associated with increased leaf size. Despite the general similarity between the two populations, and that they have one parent in common, there are numerous differences suggesting background-specific effects. Analysing multiple populations is crucial in determining the range of genetic architectures controlling these complex traits.

Increased endopolyploidy *per se* is not sufficient to drive leaf growth as evidenced by perturbation of cyclin D expression. CYCD3 regulate the timing of the transition to endocycles but knockouts do not display increased leaf area (Dewitte *et al.*, 2007; this study). Neither an endopolyploidy QTL located close to cyclin D5 nor modulation of cyclin D5 gene expression was reported to affect leaf area (Sterken *et al.*, 2012). Although the QTL on chromosome 4 identified in this study may not be identical to the cyclin D5 proximal QTL, they also do not affect leaf area. Taken together, these data support the suggestion that increased leaf growth might actually drive endoreduplication (Massonnet *et al.*, 2011) and the identification of the QTL on chromosome 5, therefore, should provide interesting insights into the interaction between leaf growth and endoreduplication.

This paper proposes that endopolyploidy represents an alternative life strategy for controlling the plasticity of organ size in *Arabidopsis* exposed to UV-B stress. The gradient of solar UV-B intensity is strongly predictive for variation in the level endopolyploidy but population structure is also particularly marked along a similar trajectory and this presents a serious confounding factor. The genetically unstructured populations (as represented by the two RIL populations) allowed this work to critically evaluate the contribution of population structure to the observed linkage between endopolyploidy and leaf area, leading the conclusion that while some QTL contribute significantly to both traits, others do not. An alternative explanation for the adaptive significance of endopolyploidy variation is that it allows for maintenance of organ growth when growth based on increased cell number is either less advantageous or becomes impaired under stressful conditions.

Previous work by the present study group reported the involvement of the UV-B photoreceptor, UVR8, in the regulation of the classic UV-B leaf expansion inhibition response (Wargent *et al.*, 2009), which demonstrated a compensatory increase in epidermal cell size in a UVR8-dependent manner was a strategy employed by leaves to compensate for a non UVR8-dependent reduction in cell number in response to UV-B in *Arabidopsis*; in addition, UVR8 was required for normal endocycle function in response to UV-B, i.e. the *uvr8* mutant displayed reduced ability to accumulate higher ploidy level cell counts under UV-B. The current work’s new observation of the high UV-B tolerance displayed by the double loss-of-function cyclin D mutant *cycd3;1/3;2* demonstrates the protective effects of high endopolyploidy against routine environmental stresses such as UV radiation, a finding complemented by the correlation between high endopolyploidy and ambient UV-B levels. At the same time, additional strategies clearly exist for plant adaptation to UV-B (e.g. pigment production). Accumulation of secondary metabolites to screen out potentially harmful wavelengths from reaching the inner leaf is a much-studied component of the UV response (Rozema *et al.*, 2002; Stracke *et al.*, 2010) and, in natural populations, a complex interaction of constitutive (i.e. noninducible) and inducible pigmentation form lines of defence against excess radiation. Little is known regarding the regulation of trade offs in plants regarding constitutive versus induced protection to UV radiation, but the findings suggest that inducible changes in the endocycle (i.e. during UV-B exposure) do make important contributions to UV tolerance compared with constitutive protection. It is possible that the endocycle may play a regulatory role within sunscreening metabolism (Vlieghe *et al.*, 2007), but other authors have already clearly shown that there is no significant difference in UV pigmentation following UV exposure of wild-type and lines with increased endopolyploidy, despite observed increased tolerance to UV-B (Hase *et al.*, 2006).

Endoreduplication in *Arabidopsis* leaves is also coupled with cellular differentiation. It is possible that the enhanced UV tolerance observed is due to aspects of cellular differentiation that have not been investigated. Other responses to UV exposure, such as generation of reactive oxygen species (ROS) (Hideg *et al.*, 2013) or enhanced DNA repair (Radziejwoski *et al.*, 2011), may also contribute to tolerance. However, taken together, the current findings support an emerging model for leaf size variation that exploits different tolerance mechanisms whose relative importance depends on evolutionary history as well as environmental conditions. Elucidating the genetic and environmental basis of leaf size variation in *Arabidopsis* will provide a useful platform to understand the relationship between growth and stress responses at multiple levels.

## Supplementary material

Supplementary data are available at *JXB* online.

Supplementary Fig. S1. Progression of endoreduplication through leaf development in Kondara and Col-0

Supplementary Fig. S2. Frequency distribution of the studied traits in Ler-Kondara RIL population

Supplementary Fig. S3. Frequency distribution of the studied traits in Kondara-Br-0 RIL population

Supplementary Fig. S4. Allelic values of significant markers for leaf area, 2C, and 32C in Ler-Kondara and Kondara-Br0 RIL populations

Supplementary Table S1. Main geographic characteristics and morphological and cellular data of the *Arabidopsis* strains studied

Supplementary Table S2. Principal components analysis

Supplementary Table S3. Spearman rank correlations between the traits studied in the Kondara-Br0 and Ler-Kondara RIL populations

Supplementary Table S4. QTL identified for the traits studied in the Ler-Kondara and Kondara-Br0 RIL populations

Supplementary Table S5. Epistatic interactions identified for the traits studied

Supplementary Table S6. ANOVA for UV responses

Supplementary Data
