# Supplementary material for: Endopolyploidy as a potential alternative adaptive strategy for Arabidopsis leaf size variation in response to UV-B
Source: J Exp Bot. 2014 Jan 27;65(10):2757–66. doi: 10.1093/jxb/ert473 (PMC4047990; doi:10.1093/jxb/ert473)
Supplement: Supplementary Data [file supp_65_10_2757__index.html]

Endopolyploidy as a potential alternative adaptive strategy for Arabidopsis leaf size variation in response to UV-B — Endopolyploidy as a potential alternative adaptive strategy for Arabidopsis leaf size variation in response to UV-B — Supplementary Data 

# Endopolyploidy as a potential alternative adaptive strategy for *Arabidopsis* leaf size variation in response to UV-B

## Supplementary Data

Data files

**Files in this Data Supplement:**

- Supplementary Data - Supplementary Data
