# Supplementary material for: Endopolyploidy as a potential alternative adaptive strategy for Arabidopsis leaf size variation in response to UV-B
Source: J Exp Bot. 2014 Jan 27;65(10):2757–66. doi: 10.1093/jxb/ert473 (PMC4047990; doi:10.1093/jxb/ert473)
Supplement: Supplementary Data [file supp_ert473_jexbot111476_file001.pdf]

Accession	Stock[a]	Code	Country of collection	Population	Latitude °North	Longitude	Altitude (m)	Leaf area (cm^2)	Cell Number[2]	Cell size (μm^2)	2C (%)	4C (%)	8C (%)	16C (%)	32C (%)	64C (%)
Cvi-0	NASC	N22614	Cape Verde Islands	Cape Verde Islands	16.0	-24.0	1100-1200	2.85	49926	5783.3	14.34	19.69	19.77	34.33	11.09	0.78
Ct-1	NASC	N22639	Italy	Catania	37.3	15.0	1-100	3.14	55778	5672.0	12.64	17.74	26.02	36.77	5.77	0.66
Kondara	NASC	N22651	Tajikistan	Pamiro-Alay	38.4	68.5	1000-1100	3.11	37899	8394.8	14.27	21.95	13.09	19.10	25.39	5.53
Sorbo	NASC	N22653	Tajikistan	Pamiro-Alay	38.4	68.5	2100-2200	2.33	33056	7257.0	16.00	21.85	14.04	28.64	18.12	1.17
Ts-5	NASC	N22648	Spain	Tossa de Mar	41.3	3.0	1-100	1.92	28631	6732.9	15.51	23.12	25.45	32.30	3.63	0.00
Kin-0	NASC	N22654	USA	Kindalville, MI	44.5	-85.4	U	1.54	26385	5878.2	12.94	16.38	18.94	40.10	10.75	0.88
Ag-0	NASC	N22630	France	Argentat	45.0	1.3	1-100	1.93	30145	6438.9	15.97	20.68	29.47	30.63	3.25	0.00
Br-0	NASC	N22628	Czech Republic	Brno (Brunn)	49.0	16.3	100-200	2.26	37034	6152.5	13.12	16.85	14.53	39.25	14.35	0.72
Kz-1	NASC	N22606	Kazakhstan	KZ	49.5	73.1	U	2.03	33047	6320.3	13.66	18.84	19.03	33.47	14.23	0.78
Kz-9	NASC	N22607	Kazakhstan	KZ	49.5	73.1	U	2.19	36944	6128.7	15.89	16.20	15.97	35.18	15.70	1.06
Mz-0	NASC	N22636	Germany	Merzhausen/Ts.	50.3	8.3	400-500	0.84	16148	5282.4	17.67	20.21	39.40	20.57	2.15	0.00
Col-0	NASC	N22625	Central Europe*	Columbia	52.4	15.2	1-100	1.47	25088	5901.2	14.37	19.44	38.88	24.80	2.50	0.00
Bur-0	NASC	N22656	Ireland	Burren	53.3	-8.0	1-100	2.44	41325	5947.3	13.65	21.32	30.55	31.62	2.86	0.00
Var-2-6	NASC	N22580	Southern Sweden	Varhallarna	55.3	14.2	U	1.68	26942	6401.6	13.00	20.64	19.26	37.31	9.28	0.52
Ms-0	NASC	N22655	Russia	Moscow	56.0	38.0	100-200	2.83	37568	7601.7	12.92	19.51	16.20	31.33	18.56	1.46
Ull2-5	NASC	N22586	Southern Sweden	Ull2	56.1	13.5	U	1.60	26618	6024.1	13.49	20.15	28.56	33.91	3.66	0.24
CS22491	NASC	N22621	Russia	Konchezero	61.4	34.2	U	2.00	26242	7696.6	11.72	19.40	13.24	29.24	24.75	1.66
				Mean	46.321	19.344	-	2.13	33458	6447.9	14.19	19.65	22.50	31.68	10.94	0.91
				Median	49.250	14.600	-	2.03	33047	6152.5	13.67	19.70	19.26	32.30	10.75	0.72
				STDEV	10.791	41.288	-	0.62	9645	832.2	1.55	1.98	8.51	5.91	7.76	1.30
				Range	45.360	158.470	-	2.30	39630	3112.4	5.96	6.92	26.31	21.01	23.24	5.53
				Min.	16.000	-85.370	-	0.84	16148	5282.4	11.72	16.20	13.10	19.10	2.15	0.00
				Max.	61.360	73.100	-	3.14	55778	8394.8	17.67	23.12	39.40	40.10	25.39	5.53

[a]NASC=Nottingham Arabidopsis Stock Centre
U= parameter not known

Table S1, related to Figure 1 and 3. Main geographic characteristics and morphological and cellular data of the Arabidopsis accessions studied. * The precise origin of Col-0 has never been defined but is likely to be Central Europe. Latitude refers to northern hemisphere coordinates. Values represent mean trait values for n=10-17 (LA), n=5 (CS, CN), and n=3 (ploidy level).

A. Arabidopsis strains

Trait	PC1	PC2	PC3
LA	-0.35	0.26	0.35
CN	-0.18	0.42	0.41
CS	-0.42	-0.23	-0.10
2C	0.17	-0.37	0.33
4C	-0.04	-0.41	0.30
8C	0.40	-0.17	0.22
16C	0.13	0.52	-0.22
32C	-0.45	-0.02	-0.21
64C	-0.42	-0.12	0.05
LAT	0.05	-0.18	-0.60
LONG	-0.29	-0.24	-0.02
Var. %	37.8	23.8	17.1
Total			78.7

B. Ler x Kondara

Trait	PC1	PC2	PC3
2C	0.23	0.35	0.43
4C	0.38	0.19	0.41
8C	0.34	-0.44	-0.02
16C	-0.40	-0.15	-0.38
32C	-0.37	0.45	-0.03
64C	-0.29	0.49	0.05
LA	-0.32	-0.25	0.41
Var. %	43.2	21.3	15.1
Total			79.6

C. Kondara x Br-0

Trait	PC1	PC2
2C	0.26	0.23
4C	0.30	0.34
8C	0.38	0.32
16C	-0.40	-0.37
32C	-0.29	-0.22
LA	-0.34	0.38
Var. %	44.8	20.8
Total		65.6

Table S2, related to Figures 3 and 4. Principal Components Analysis –Loadings and var. explained for **(A)** the *Arabidopsis* strains and **(B-C)** the two mapping populations.

A. Kondara-Br0 RIL population

LA	1						
2C	-0.27	1					
4C	**-0.10**	0.43	1				
8C	-0.34	0.27	0.43	1			
16C	0.31	-0.48	-0.63	-0.92	1		
32C	0.30	-0.27	-0.41	-0.91	0.80	1	
64C	0.25	**-0.07**	**-0.09**	-0.35	**0.18**	0.46	1
	LA	2C	4C	8C	16C	32C	64C

B. Ler-Kondara RIL population

LA	1						
2C	-0.36	1					
4C	-0.45	0.71	1				
8C	-0.34	**-0.13**	0.36	1			
16C	0.44	-0.60	-0.89	-0.48	1		
32C	0.39	**-0.10**	-0.49	-0.91	0.50	1	
64C	0.34	**-0.05**	-0.38	-0.84	0.39	0.92	1
	LA	2C	4C	8C	16C	32C	64C

Table S3. Spearman rank correlations between the studied traits in the Kondara-Br0 **(A)** and Ler-Kondara **(B)** RIL populations. All correlations are performed on untransformed data. Values in bold represent non-significant correlations, the rest are significant at the level of 0.001.

Trait	Population	Chrom.-Marker	Position(cM)	LOD‡	%Expl.¶	Effect‖
LA	L-K	2-ERECTA	25.0	4.29	15.0	-0.14
		5-SNP97	56.0	2.78	9.8	-0.11
	K-B	3-f17a931902	9.3	2.8	11.6	0.09
		5-ciw9	44.0	2.65	10.5	0.09
2C	L-K	2-ERECTA	25.0	15.52	44.8	1.73
		3-SNP114	3.0	3.21	11.5	0.88
	K-B	2-athubique	49.9	2.89	13.8	0.62
		3-f17a931902	9.3	3.61	16.4	-0.68
4C	L-K	1-CIW1	59.0	3.22	13.1	1.69
		2-SNP135	30.0	3.8	15.1	1.80
8C	L-K	2-ERECTA	25.0	2.74	10.5	-2.15
		4-FRI	4.0	2.85	11.3	2.23
		5-SNP97	56.0	3.16	12.7	2.37
	K-B	4-CIW6	37.4	2.94	14.2	-0.30
16C	L-K	2-SNP233	37.0	3.4	17.7	-3.05
32C	L-K	4-FRI	4.0	4.54	16.4	-1.75
		5-SNP97	56.0	3.39	13.3	-1.59
	K-B	4-CIW6	37.4	2.68	13.0	0.17
		5-JV65-66	56.1	2.6	11.7	0.14
64C	L-K	4-FRI	4.0	2.63	9.7	-0.14
		4-MSAT4.37	54.0	2.98	10.7	-0.15
		5-SNP97	56.0	4.12	14.7	-0.18

‡ LOD scores for each of the significant QTL identified,

¶ Percentage of phenotypic variance explained by each QTL, ‖ Additive effect.

Table S4, related to Figure 5. QTL identified for the traits studied in the Ler-Kondara (L-K) and Kondara-Br0 (K-B) RIL populations. No significant QTL were identified for the 4C and 16C in the Kondara-Br0 population.

a. Ler-Kondara

trait[†]	marker1	marker2	p-value[‡]
2C	SNP135	SNP236	0.0001
2C	SNP135	SNP193	0.0005
2C	erecta	SNP193	0.0008
2C	erecta	SNP236	0.0009
8C	FRI	SNP358	0.0000
8C	msat4.8	SNP358	0.0002
16C	SNP233	SNP358	0.0002
32C	FRI	SNP136	0.0001
32C	msat4.8	SNP358	0.0001
32C	msat4.3	SNP358	0.0001
32C	FRI	SNP358	0.0000
32C	msat4.8	SNP136	0.0000
32C	msat4.3	SNP136	0.0000
32C	msat4.8	SNP77	0.0002
32C	msat4.3	SNP77	0.0002
32C	FRI	SNP21	0.0005
64C	FRI	SNP21	0.0000
64C	FRI	SNP53	0.0001
64C	FRI	SNP358	0.0001
64C	FRI	SNP77	0.0009
64C	msat4.37	msat4.8	0.0008
64C	msat4.37	FRI	0.0005
64C	FRI	SNP136	0.0002

b. Kondara-Br0

trait	marker1	marker2	p-value
LA	athsrp54a	pls1	0.0004

[†]Traits for which significant interactions between the corresponding markers have been detected.
[‡]p-values for significant interactions established by 1,000,000 MonteCarlo simulations (threshold p=0.001).

Table S5, related to Figure 5. Marker interactions identified for the traits of endoploidy and leaf area in the (A) Ler-Kondara and (B) Kondara-Br0 RIL populations. Only the interactions above the significance threshold of p=0.001 are reported.

A. Rosette fresh weight

	SS	*df*	*MS*	*F*	*P-value*
Genotype	0.012	3	0.004	10.63	1.297E-05
UV	0.043	1	0.043	114.05	5.028E-15
Gt x UV	0.002	3	0.001	2.07	1.141E-01
Within	0.020	55	0.000		
Total	0.078	62			

B. UV-pigments (300nm)

	SS	*df*	*MS*	*F*	*P-value*
Genotype	139.69	1	139.69	397.63	4.87E-31
UV	10.62	3	3.5397	10.08	1.27E-05
Gt x UV	5.77	3	1.9227	5.47	0.001913
Within	25.29	72	0.3513		
Total	181.37	79			

C. Ploidy (≥16C)

	SS	*df*	*MS*	*F*	*P-value*
Genotype	93.97	1	93.97	11.17	0.004139
UV	1410.98	3	470.33	55.90	1.06E-08
Gt x UV	255.87	3	85.29	10.14	0.000555
Within	134.63	16	8.41		
Total	1895.45	23			

Table S6, related to Figure 6. ANOVA for UV responses.
